# Structural Features of Cytochrome *b*_5_–Cytochrome *b*_5_ Reductase Complex Formation and Implications for the Intramolecular Dynamics of Cytochrome *b*_5_ Reductase

**DOI:** 10.3390/ijms23010118

**Published:** 2021-12-23

**Authors:** Carlos Gutiérrez-Merino, Oscar H. Martínez-Costa, Maria Monsalve, Alejandro K. Samhan-Arias

**Affiliations:** 1 Department of Biochemistry and Molecular Biology, Faculty of Sciences and Instituto de Biomarcadores de Patologías Moleculares, Universidad de Extremadura, Av. Elvas S/N, 06006 Badajoz, Spain; 2 Instituto de Investigaciones Biomédicas ‘Alberto Sols’ (CSIC-UAM), Arturo Duperier, 4, 28029 Madrid, Spain; oscar.martinez@uam.es (O.H.M.-C.); mpmonsalve@iib.uam.es (M.M.); 3 Department of Biochemistry, Faculty of Medicine, Universidad Autónoma de Madrid (UAM), Arzobispo Morcillo, 4, 28029 Madrid, Spain

**Keywords:** cytochrome *b*_5_ reductase, cytochrome *b*_5_, superoxide anion radical, electron transfer, protein intrinsic dynamics

## Abstract

Membrane cytochrome *b*_5_ reductase is a pleiotropic oxidoreductase that uses primarily soluble reduced nicotinamide adenine dinucleotide (NADH) as an electron donor to reduce multiple biological acceptors localized in cellular membranes. Some of the biological acceptors of the reductase and coupled redox proteins might eventually transfer electrons to oxygen to form reactive oxygen species. Additionally, an inefficient electron transfer to redox acceptors can lead to electron uncoupling and superoxide anion formation by the reductase. Many efforts have been made to characterize the involved catalytic domains in the electron transfer from the reduced flavoprotein to its electron acceptors, such as cytochrome *b*_5_, through a detailed description of the flavin and NADH-binding sites. This information might help to understand better the processes and modifications involved in reactive oxygen formation by the cytochrome *b*_5_ reductase. Nevertheless, more than half a century since this enzyme was first purified, the one-electron transfer process toward potential electron acceptors of the reductase is still only partially understood. New advances in computational analysis of protein structures allow predicting the intramolecular protein dynamics, identifying potential functional sites, or evaluating the effects of microenvironment changes in protein structure and dynamics. We applied this approach to characterize further the roles of amino acid domains within cytochrome *b*_5_ reductase structure, part of the catalytic domain, and several sensors and structural domains involved in the interactions with cytochrome *b*_5_ and other electron acceptors. The computational analysis results allowed us to rationalize some of the available spectroscopic data regarding ligand-induced conformational changes leading to an increase in the flavin adenine dinucleotide (FAD) solvent-exposed surface, which has been previously correlated with the formation of complexes with electron acceptors.

## 1. The Electron Transfer from the Cytochrome *b*_5_ Reductase to Multiple Acceptors and Implication in Reactive Oxygen Species Formation

Flavoproteins can be categorized into three main types: oxidases, dehydrogenases, and monooxygenases. The distinction between them has been made based on the use of a semiquinone flavin radical as part of the catalytic mechanism, which is present in dehydrogenases but absent in monooxygenases and oxidases [1]. Early on, based on kinetic studies using stopped-flow techniques, Prof. Philipp Sttritmatter suggested the electron transfer mechanism of the cytochrome *b*_5_ reductase (C*b*_5_R) [2]. This electron transfer process depends on the rapid formation of a complex with NADH, which is rapidly oxidized and meets the requirements for an intermediate generation in the catalytic reaction. In addition, Prof. Sttritmatter [2] showed evidence of the existence of, at least, one intermediate involved in flavin reduction and a second implicated in flavin reoxidation, both part of a complete oxidative cycle. The reaction of NADH with the oxidized enzyme is fast and occurs in less than 2 ms, prompting the formation of a stable complex. Since the oxidation of the reduced enzyme complex is faster than the reduction in the flavoprotein, the oxidized flavoprotein and the reduced pyridine nucleotide complex are the predominant forms during the catalytic turnover. In this reaction, one-electron oxidation can be measured, leading to the formation of a flavin semiquinone radical which can be spectroscopically measured [3]. The formation of the flavin semiquinone radical was reported to be independent of the substrate used: cytochrome *b*_5_ (C*b*_5_) or ferricyanide. Later, it was shown that C*b*_5_ presence increases the binding constant of oxidized nicotinamide adenine dinucleotide (NAD^+^) of the quaternary complex [4]. The pyridine nucleotide remains bound to the enzyme throughout the entire oxidation process. The final oxidation step is the rate-limiting step in the catalytic cycle. NAD^+^ slowly autoxidizes and disproportionates the FAD semiquinone [4]. As shown later by pulse radiolysis techniques, one electron can react with NAD^+^, yielding NAD^•^ [5]. The electron can be transferred to the NAD^+^-bound oxidized enzyme to form the blue and red semiquinone or a mixture of the two forms of the enzyme, where p*K*_a_ value of this flavin radical was approximately 6.3 [5]. Very few changes in the electron transfer mechanism have been shown since this mechanism was suggested. The involvement of one tyrosine residue and SH groups was suggested early on to be implicated in the catalytic processes, although no specific amino acid residues were identified [2].

At that time, the role of free radicals produced by enzymes in diseases had not been explored. Notably, the transient formation of a flavin radical in the electron transfer mechanism suggested that reactive oxygen species could be formed within the catalytical mechanism of the C*b*_5_R. When oxygen is present in the media, reactive oxygen species production can be catalyzed by flavins and flavoproteins [1,6]. In the case of flavoproteins, oxidases, hydroxylases, and dehydrogenases, they all rapidly or slowly react with oxygen in a flavin-semiquinone-dependent or -independent way leading to the formation of free radicals [1]. However, this might be modulated by endogenous electron acceptors or biological molecules present in the subcellular compartments where C*b*_5_R localizes.

C*b*_5_R is a pleiotropic oxidoreductase enzyme that donates electrons to many redox acceptors aside from oxygen. This enzyme modulates many metabolic pathways by electron transfer to biological electron acceptors. This enzyme has different isoforms, but the catalytic domain is highly conserved between them [7]. The presence of a soluble isoform C*b*_5_R was described in erythrocytes as the main responsible for the enzymatic recycling of methemoglobin [8]. The membrane isoform of C*b*_5_R has a domain deeply inserted within the lipid bilayer that anchors this enzyme to different subcellular membranes. The membrane isoform is located in the outer leaflet of the endoplasmic reticulum (ER), the mitochondrial outer membrane (MOM), and the plasma membrane [9]. The C*b*_5_R membrane isoform is made up of 300 amino acid residues comprising a soluble domain, which is formed by 275 residues oriented toward the cytosol, and the N-terminal tail of 24 residues anchoring the protein to the membrane [10]. This soluble domain is similar to the erythrocyte soluble isoform that only presents the C-terminal soluble domain [11]. The membrane isoform is present in almost all mammalian cells as a membrane-bound protein, including erythrocytes. The molecular mechanism for the distribution of the reductase across different subcellular locations is not completely clear. The topography of the membrane domain allows the membrane isoform to penetrate deeply into the lipid bilayer. Notably, the mechanism for spreading along the membrane and how the enzyme confines to the cytoplasmic leaflet in a hairpin conformation have been questioned [11,12].

### 1.1. The Endoplasmic Reticulum Cb_5_R

C*b*_5_R membrane isoforms have N-terminal domains that anchor the soluble domain into the microsomal membrane [10,11], and the MOM [13]. A single point mutation demonstrated that the same gene product localizes the protein in two different subcellular compartments [14]. The primary function of the C*b*_5_R located at the ER is the electron transfer to desaturases and participation in detoxification pathways in conjunction with C*b*_5_ and cytochrome P450s [15]. Direct mutagenesis experiments showed that myristylation was key for the reductase targeting the MOM, while the non-myristoylated mutant was only found in the ER [16]. The first seven residues of the membrane C*b*_5_R isoform translation product, which includes the methionine, constitute the myristylation signal [17]. The codons that target the protein for myristylation can be excluded or included in the transcript in a tissue-specific manner [11]. The biological function of C*b*_5_R in membranes is also facilitated by the membrane isoform of cytochrome *b*_5_ (C*b*_5_), which also possesses a membrane-binding domain in the protein structure and a water-soluble domain that interacts with the cytosol. This is particularly relevant for many metabolic functions of C*b*_5_R because C*b*_5_ is an electron carrier of many membrane-bound enzymes of the lipid metabolism, such as cytochrome P450 monooxygenases and membrane redox chains (reviewed in [15]). It is noteworthy that both soluble and membrane-bound C*b*_5_ isoforms modulate the activity of membrane cytochrome P450 monooxygenases involved in xenobiotic metabolism [18]. Additionally, soluble C*b*_5_ can act as an electron acceptor of the reductase present in membranes [19]. Since both cytochrome P450 monooxygenases and C*b*_5_R can produce reactive oxygen species (ROS) in the absence of electron acceptors [15,20], C*b*_5_ can be regarded as an antioxidant protein that prevents excessive intracellular ROS production during drug detoxification [18]. This effect can also be considered as derived from microsomal cytochromes P450 requirement of two electrons and two protons for the oxidation of substrates. Although the two electrons can be provided by cytochrome P450 reductase, the second electron can also be donated by C*b*_5_, leading to a more rapid protonation of the anionic ferric hydroperoxy–heme intermediate of P450 [21]. This fosters the C*b*_5_ stimulatory effect through a more efficient coupling of the system components.

### 1.2. The MOM Cb_5_R

Interestingly, in *Saccharomyces cerevisiae*, mitochondrial C*b*_5_R sorts into different mitochondrial compartments, due to 40 amino acid residues at the N-terminal end of the membrane isoform C*b*_5_R not conserved in other flavoenzymes. Two forms of 34 kDa and 32 kDa have been detected in this yeast locating at the MOM and in the intermembrane space, respectively [22]. The 34 kDa form has a putative 21 amino-terminal matrix terminal signal, followed by 21 uncharged hydrophobic residues that are transported into the intermembrane space, which is cut by the inner membrane protease 1 to generate the 32 kDa form of the reductase. The function of the intermembrane space isoform present in yeast is not clearly defined, although it was early suggested to be involved in the electron transfer from external NADH to cytochrome c, thereby mediating antimycin-insensitive, energy-coupled oxidation of external NADH by yeast mitochondria [23].

The function assigned to the mammalian C*b*_5_R located at the MOM depends on its coupling with other proteins. Some studies have associated the reductase present at this location with the formation of a complex with the mitochondrial amidoxime reducing component and the outer mitochondrial C*b*_5_. This complex is in charge of the activation of prodrugs containing an amidoxime structure and detoxification pathways [24]. A very recent role of C*b*_5_R located at the MOM has been suggested in endothelial cells expressing NADPH oxidase 4 (NOX4). As a mitigator of inflammatory activation, C*b*_5_R would reduce the NOX4-dependent production of H_2_O_2_ via the reduction of ubiquinone (CoQ), a substrate of C*b*_5_R. [25]. Yuan et al. (2021) suggest that the electron transfer through CoQ plays a role in shifting the outer membrane NOX4 superoxide anion radical (O_2_^•−^) production to H_2_O_2_ [25]. The presence of a transmembrane domain in charge of H_2_O_2_ production associated with the E-loop [26] suggests that indeed this enzyme NOX4 might work as a redox-signaling transducer. Additionally, NOX4–C*b*_5_R complexes seem necessary for proper coupling between both enzymes, because excessive ubiquinol oxidation has been reported to generate H_2_O_2_ and O_2_^•−^ [27,28,29,30]. Notably, the importance of correct clustering of C*b*_5_R with other proteins or associated with a correct membrane environment has been pointed out in the plasma membrane of neurons [19,31,32,33].

### 1.3. The Plasma Membrane Cb_5_R

A proposal has been made to rationalize the location of the membrane isoform at the plasma membrane using erythrocyte membranes—namely, an N-terminal-extended erythroid polypeptide with an N-terminus 12 uncharged reticulocyte-specific residues, in addition to 17 residues of the membrane that anchors the myristoylated reductase [11,34,35]. The membrane isoform of C*b*_5_R is the major NADH consuming enzyme at the plasma membrane of vesicles derived from rat brain synaptosomes (SPMV) and the neuronal plasma membranes [19,31]. We have reported that C*b*_5_R accounts for 80% of the NADH oxidase activity, by analyzing the activation data in the presence of cytochrome activation and soluble C*b*_5_ (3–4 μM) [19,31]. C*b*_5_ regulates this enzyme function [19]. The amount of C*b*_5_ present in SPMVs is not at saturation since the NADH oxidase activity is stimulated by supplementation with soluble C*b*_5_, which elicits a three-fold activation on this activity [19]. C*b*_5_R location at the plasma membrane lipid rafts suggests a role of this enzyme in cholesterol metabolism. A role regarding an in situ cholesterol formation at this location should not be discarded based on C*b*_5_ functions in the last steps of cholesterol synthesis [9,15]. We have reported that C*b*_5_R/C*b*_5_ can be a source of reactive oxygen species using the purified enzyme, biological membranes, and culture cells [28,31,33]. Cholesterol-rich plasma membrane sub microdomains have been suggested to be a major extramitochondrial O_2_^•−^ source in cerebellar granule cells cultures [32].

In addition, our laboratory has described that the NADH oxidase activity of SPMVs is similarly stimulated in the presence of horse heart Cyt *c* with an IC_50_ of 6 μM [36]. The Cyt *c* stimulated NADH oxidase activity of SPMV can be inhibited by the addition of antibodies against C*b*_5_R to the assay [19]. This fact correlates with the ability of recombinant C*b*_5_R to use also Cyt *c* as an electron acceptor and a role of C*b*_5_R performing this function in membranes [33]. The activity of the membrane C*b*_5_R bound to the neuronal plasma membrane can be modulated by changes in cytosolic levels of C*b*_5_ and Cyt *c* in the low micromolar concentration range. However, this activity is about three times more sensitive to C*b*_5_ than to Cyt *c* [19,36]. The similar dissociation constant of the C*b*_5_:C*b*_5_R complex with respect to that of the Cyt *c*:C*b*_5_R complex (0.4–0.5 μM) [33,37] correlates with other possible endogenous ligands of Cyt *c* that might be present in these membranes.

We postulated a protective role of C*b*_5_R by its ability to reduce oxidized Cyt *c* [33], a widely recognized pro-apoptotic factor that is needed for caspases activation [38,39] and cardiolipin-induced Cyt *c* peroxidase activity [40], early events in apoptotic cell death [33].

### 1.4. Antioxidants Recycling Activity of Cb_5_R and the Importance in Cellular ROS Balance

C*b*_5_R function was early revealed as an enzyme in charge of antioxidants recycling [39,41,42,43]. Tocopherol is a lipophilic antioxidant important for the stability of lipids and phospholipids placed within the (sub)cellular membranes [44]. α-tocopherol has a leading role against lipid peroxidation, and the redox reaction with other cellular antioxidants such as GSH, CoQ, and ascorbate have been directly related to membrane protection and cell function maintenance [45,46]. Tocopheroxyl radical is generated upon the reaction of α-tocopherol with lipid hydroperoxides. Antioxidants can react with the tocopheroxyl radical to recycle it [45,46]. Additionally, those cellular elements capable of reducing radicals are key to restoring tocopherol’s antioxidant power [47]. C*b*_5_R and other proteins with quinone reductases activity, such as the plasma membrane NADH quinone oxidoreductase (NQO1) and the mitochondrial NADH-quinone oxidoreductase of mitochondrial electron transfer complex I, have been postulated to present this function. C*b*_5_R reduces CoQ at the expense of soluble NADH through a one-electron reaction mechanism. NQO1 also reduces CoQ through a two-electron reaction mechanism using both NADH and NADPH [48]. This difference between both systems may be significant under oxidative stress conditions since the formation of the semiquinone radical could lead to the production of O_2_^•−^ upon by its reaction with molecular oxygen, as reported, by the mitochondrial redox chain and, also found by C*b*_5_R reacting with CoQ mimetics [28]. The semiquinone radical and possible deleterious side reactions can be avoided by C*b*_5_R recycling the semiquinone radical, reducing the radical rather than reducing it after generation of the oxidized quinone. Semiquinone generation has been proposed to be key for the formation of ROS [49]. This is also supported by experiments showing that the formation of CoQ semiquinone radical and recycling of vitamin E homologs are O_2_^•−^-dependent reactions, which agree with the ability of C*b*_5_R to produce O_2_^•−^ [20,28,31,33]. In addition, the formation of ROS in biological systems depends on the reaction between ubiquinol and electron partners accepting two electrons leading to the formation of oxidized CoQ. The proximity of systems recycling CoQ in two electron-reducing pathways and the slower reaction rate between C*b*_5_R and the semiquinone radical vs. reaction with other molecules leads to radical reactions in which C*b*_5_R cannot participate. Additionally, a key molecule in semiquinone reduction is soluble ascorbate [50]. Ascorbate radical and ubiquinol are products of this reaction. Ascorbate radical can be recycled by the NADH: ascorbate radical reductase activity of C*b*_5_R [41,42,50]. The reaction rates of O_2_^•−^/ascorbate and O_2_^•−^/ascorbate radical are reported: 5 × 10^4^ M^−1^ s^−1^ and 2.6 × 10^8^ M^−1^ s^−1^, respectively [51]. These values are close to the ones reported for the reaction rate of O_2_^•−^ with superoxide dismutase [52].

## 2. Structural Features of Soluble C*b*_5_R Helps to Rationalize the Electron Transfer Processes Using NADH as a Substrate

### 2.1. FAD-Binding Domain of Cb_5_R

**Figure 1:**
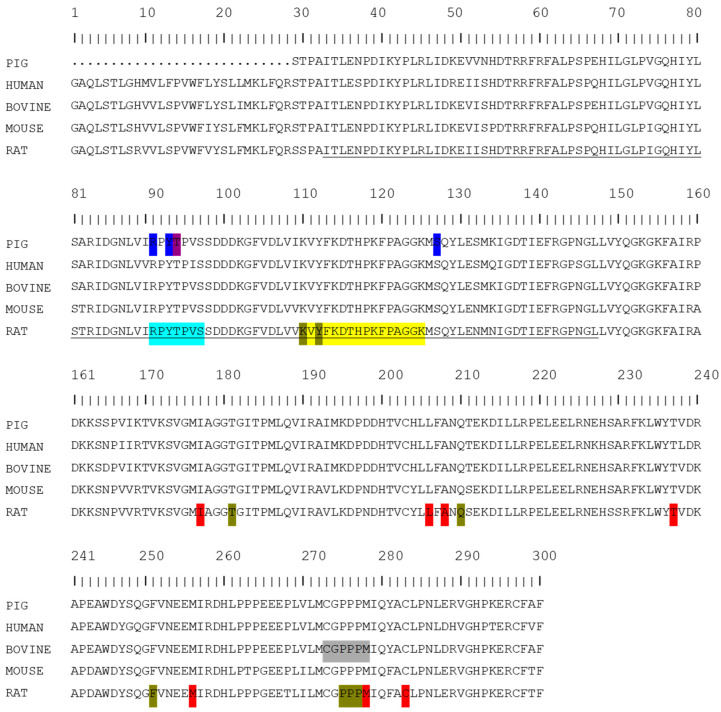
Alignment of C*b*_5_R isoforms from different sources. Pig (Unitprot: P83686), human (Unitprot: P00387), bovine (Unitprot: P20070) and rat (Unitprot: P20070). Amino acid residues 33–147 located at the amino-terminal side of the FAD-binding domain (underlined sequence); residues 110–125 forming a loop in the FAD-binding domain (yellow background); the conserved motif “91RxYTSxxSN97” (light blue background); location of the R91, Y93, and S127 (dark blue background); location of the T66 of the pig enzyme implicated in the formation and stability of the NAD+–FAD semiquinone complex during NADH turnover which correlate with T94 in the human isoform (purple background); location of the residues forming the plateau where the NAD^+^ group sets and in which the adenine ring and the diphosphate group packed between the side chain residues of these amino acid residues (golden background); Proposed residues that could alter the hydrophobic environment formed by the residues I177, L206, A208, T237, M256, M278, and C283 affecting the binding of NAD^+^ (red background); suggested sequence ^273^CGPPPM^278^ to be critical for the correct orientation of the nicotinamide moiety with the flavin for efficient hydride transfer (grey background).

Several conserved motifs have been observed in C*b*_5_R’s primary structure in comparison with other FAD-containing proteins, which have been implicated in the binding of the flavin group, such as “RxY^T^_S_xx^S^N” [53]. In rat C*b*_5_R, the FAD-binding domain is comprised of amino acid residues 33–147 located at the amino-terminal side (Figure 1, underlined sequence) [54]. The loop formed by residues 110–125 (Figure 1, yellow background) contributes to most of the interactions between the FAD-binding domain of the reductase and the adenine dinucleotide moiety of the FAD group in which water mediates the interaction [54]. The conserved motif “^91^RxY^T^_S_xx^S^_N_^97^” has been proposed to rule flavin binding to the apoprotein in the flavin transhydrogenase superfamily of oxidoreductases, in which the NADH:*Cb_5_R* is included (Figure 1, light blue) [55]. Nishida and Kimura proposed that R63, Y65, and S99 residues of the motif of the pig soluble enzyme (R91, Y93, and S127 residues in Figure 1, dark blue) are important in the flavin coordination through the hydrogen bonding of the phosphate and ribityl moieties of the cofactor [56]. Later, Barber’s research group showed that indeed R91 was not essential for flavin binding but participated in tethering the ADP moiety of the FAD cofactor by H bonding to the protein [57]. Moreover, a role of T66 of the pig enzyme (T94 in Figure 1, purple) in the formation and stability of the NAD^+^–FAD semiquinone complex during NADH turnover was shown [58]. Later, the role of P92 and Y93 residues of the rat C*b*_5_R was reevaluated, concluding that these residues are not important for FAD incorporation into the apoprotein. Notably, mutagenesis experiments showed that Y93 residue contacts with the FAD group and modulates spectroscopical, catalytic, and thermodynamic properties of the FAD cofactor [55]. This suggests that these residues are important for the redox modulation of the enzyme by C*b*_5_ [37]. Moreover, methemoglobinemia is reported when mutations in C*b*_5_R’s residues, V105M and M126V, are present. These mutations induce perturbations of the FAD-binding domain [54] since the side-chain atoms of M126 are housed by the hydrophobic pocket formed by residues: L80, T82, V89, Y93, Y129, I139, and the hydrophobic atoms of R91 [54].

### 2.2. The NADH-Binding Pocket and Hydride Transfer to the Flavin Group of the Reductase

Comparative analysis with other FAD-binding proteins allowed the identification of some motifs in the primary sequence of C*b*_5_R that are involved in the interaction with reduced pyridine nucleotide, and, also, in the selectivity of the interaction between NADH/NADPH and the enzyme such as “GxGxxP” and “CGxxxxM” [53]. In addition, an NADH-binding lobe was proposed by Barber’s research group, with a function in setting NAD^+^ to the plateau formed by proline residues 275–277, in which the adenine ring is packed between the side chain residues, in parallel to F251 and P277 (Figure 1, golden background). Moreover, the NADH diphosphate group packs against P275 and the oxygen atoms from the phosphate group from H^+^-bonds with Q210, T181, Y112, and K110 [54] (Figure 1, golden background). Experiments with the rat isoform indicate that mutations in V253 residue affect the NAD^+^ binding by altering the hydrophobic environment formed by the residues I177, L206, A208, T237, M256, M278, and C283 [59] (Figure 1, red background). The sulfhydryl group of C245 (C283 of the rat C*b*_5_R) also forms Van der Waals contact with the nicotinamide C3 atom at the *si*-face [60]. This interaction is considered to alter the redox potential of the NAD^+^ upon binding to the enzyme. C273 was observed to form part of a sequence ^273^CGPPPM^278^, which was suggested to be critical for the correct orientation of the nicotinamide moiety with the flavin for efficient hydride transfer [61] (Figure 1, grey background).

The second conserved motif among pyridine nucleotide-binding FAD proteins is “GxGxxP”, which is located in residues between the G180-P185 residues of the carboxyl-terminal lobe of rat C*b*_5_R and a recognized function in the binding of reduced pyridine nucleotides [62]. The importance of the G179 residue preceding this motif was shown by direct mutagenesis since its mutations induce changes in both the adequate NADH/NADPH selectivity and NADH binding and efficient hydride transfer [62]. Some catalytic properties are shared between dehydrogenases that use NADPH as a substrate. Enzymes that present a dehydrogenase activity accept two electrons from the substrate to carry electrons to a final metalloprotein, normally a heme or iron-dependent protein that acts as an electron acceptor [63]. Iyanagi suggested that for the case of C*b*_5_R, its dehydrogenase activity is associated with a direct hydride ion transfer to the flavin group [63]. The two-electron reduced enzyme−NAD^+^ complex (E-FADH^−^−NAD^+^) then transfers two electrons to two one-electron acceptors one by one, after which the reduced enzyme returns to the oxidized state.

## 3. C*b*_5_-Interacting Domain of C*b*_5_R

The topography of the human C*b*_5_/C*b*_5_R-interacting domain has been reported in [37]. The results obtained in NMR experimental studies and docking simulation allowed the identification of the C*b*_5_R amino acids more directly involved in the interaction with C*b*_5_—namely, K41, Y79, N87, L88, V89, V90, R91, P92, F120, G123, K125, R142, S145, L147, P160, D161, K162, K163, M272, P276, P277, R279, Q280, Y281, L284, T294, F298, V299, and F300 [37]. In addition, the results reported in Samhan-Arias et al. [37] allowed the prediction of two salt bridges and four H-bond pairs formation in the C*b*_5_/C*b*_5_R interacting. The predicted salt bridges are K162(C*b*_5_R):E42(C*b*_5_) and K125(C*b*_5_R):E48(C*b*_5_), and the predicted H bonds are V89/V90(C*b*_5_R):E47(C*b*_5_), Y281(C*b*_5_R):D64(C*b*_5_), Y79(C*b*_5_R):P44/H43/Q53(C*b*_5_) and L88(C*b*_5_R):E48/R51(C*b*_5_).

### Electron Transfer from the Flavoprotein to Cb_5_

Several research groups have studied the electron transfer kinetics to C*b*_5_ from the reduced C*b*_5_R. In general, the electron transfer reactions between flavins/flavoproteins and electron acceptors can follow one or two electron-dependent pathways [64]. Both electron transfer mechanisms and can be distinguished spectroscopically by analyzing the UV–visible spectrum of the flavin group [1,65]. The oxidized flavin is yellow, with maximum intensity at 370 and 450 nm, and the hydroquinone form (fully reduced) has very weak broad spectra [65]. The unprotonated form of red anionic semiquinone has a maximum band of 370 nm. However, if N(5) of FAD is protonated, a strong band at 570 nm resulting from the blue neutral semiquinone formation is observed [65]. The formation of one or the other redox state in flavoproteins depends on the protein microenvironment because hydrophobicity and proximal charged amino acids might be key variables regulating the flavin redox state. The C*b*_5_R’s flavin semiquinone stabilization is affordable when a more positive reduction potential for the first single-electron transfer step is found over the second single electron transfer step [65].

**Figure 2:**
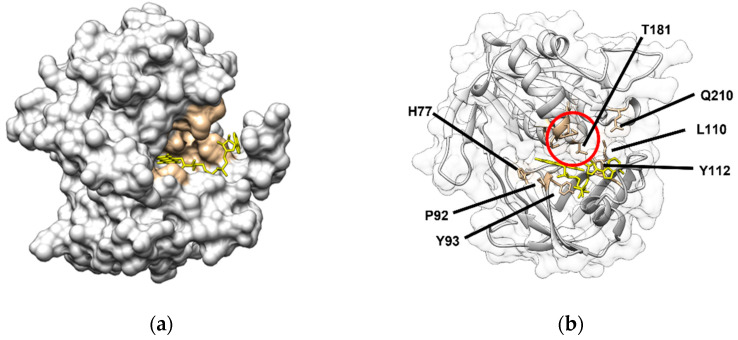
FAD protruding site of C*b*_5_R (PDB: 1UMK) and residues involved in NADH binding. Surface representation of the human C*b*_5_R structure obtained by crystallography (PDB:1UMK) and location of the FAD (represented as yellow sticks) protruding site is shown in panel (**a**). Residues interacting with the diphosphate groups and the adenine ring of NAD^+^, as reported, are labeled in brown. The same representation is shown in panel (**b**), where the surface is transparent, and the residues interacting with NAD^+^ (brown), and the backbone can be viewed (grey). The red circle labels the ^273^CGPPPM^278^ motif, which forms the NAD^+^-binding surface.

**Figure 3:**
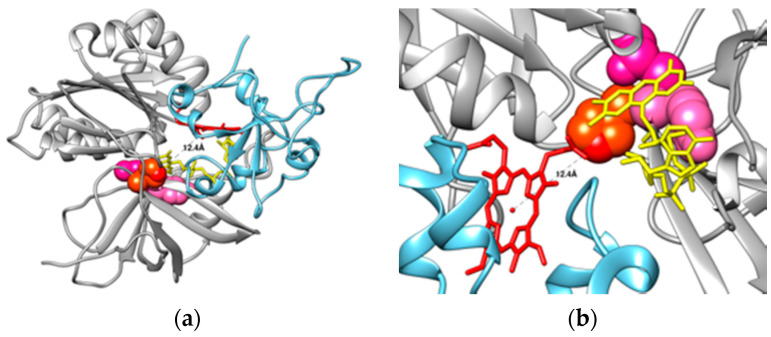

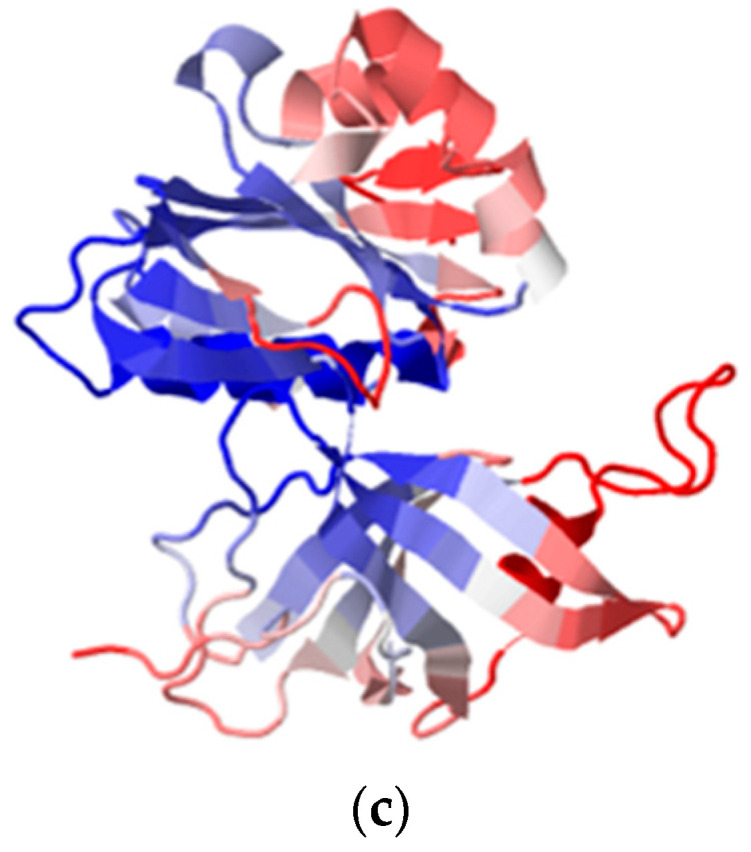
Representation of the C*b*_5_:C*b*_5_R complex model obtained by docking and representation of C*b*_5_R areas that are susceptible for mobility through intraprotein dynamics. The C*b*_5_:C*b*_5_R complex model has been previously published [37]. C*b*_5_R’s backbone is shown in grey color, with side-chain residues of Y93, T94, and H77, which are represented as light pink, dark pink, and red-colored balloons, respectively, the flavin group depicted as sticks in yellow interact with the heme group (represented as red sticks) of C*b*_5_ backbone (represented in light blue color). Also, a distance of 12.4 Å from the iron heme group of C*b*_5_ to NE2 of H77 was measured and labeled, as shown in panel (**a**). The zoomed area where a distance of 12.4 Å was found between the iron heme group of C*b*_5_ and NE2 of H77 is shown in panel (**b**). The chain corresponding to C*b*_5_R (1UMK) from our model for the C*b*_5_:C*b*_5_R complex was submitted to the dynOmics portal, as indicated in the text, and a figure representing C*b*_5_R was obtained, where the size of fluctuations by the lowest frequency (slowest) two Gaussian network model (GNM) modes (blue: almost rigid; and red: highly mobile), as indicated by the color bar under the diagram window) (panel (**c**)).

Based on crystallographic data, the electron pathways from the reductase to C*b*_5_ have been suggested to directly proceed following the hydrogen bond paths (FAD-N5⋯Y65/T66⋯H49⋯C*b*_5_) [66]. These residues are labeled as Y93, T94, and H77 in Figure 2, panel b (Y65, T66, and H49 in the manuscript). Additionally, this is supported by the observations showing a stabilization of the N5 atom of the isoalloxazine ring of FAD by hydrogen bonding to Y93 and T94 and the highly conserved H77. The importance of H77 has been shown by mutagenesis experiments of this residue. Y93, T94, and H77 residues are in the backside of the crevice, where the FAD binds to C*b*_5_R (Figure 2). Notably, the C*b*_5_-binding site that we have described by docking analysis indicates that the heme group is approximately at a 12 Å distance from the H77 (Figure 3a,b). It might suggest a possible role of these residues in the bifurcation of electrons to different acceptors or to increase the half-life of the flavin radical species if they are generated in an oxidative environment.

## 4. Flavin Fluorescence and Structural Alterations by Complexes Formation with Electron Acceptors

C*b*_5_ addition to the reductase induced an increase in the C*b*_5_R flavin autofluorescence, compared with the free FAD cofactor [37]. This effect was not associated with the release of the flavin group from the protein. The increase in the reported fluorescence intensity by the complex formation with C*b*_5_ suggests that C*b*_5_R undergoes a conformational change induced by the presence of electron acceptors of this enzyme. Therefore, we used these changes to characterize the complex formation between the soluble isoforms of C*b*_5_R and C*b*_5_ [37]. From the dependence upon C*b*_5_ of the increase in C*b*_5_R’s flavin autofluorescence, we calculated a *K*_d_ value of 0.5 μM C*b*_5_. The stoichiometry for the complex formation between these proteins is 1:1 [37]. As indicated above, these changes in fluorescence monitor structural alterations in the binding site of FAD and correlate with changes in the redox potential of C*b*_5_R upon binding of C*b*_5_. The reported redox potential of C*b*_5_R was to −196 ± 8 mV and shifted to −239 ± 4 mV upon complex formation with C*b*_5_ [37]. Indeed, this result is in good agreement with the results obtained with other flavoproteins, which have led to the conclusion that flavoproteins can modulate their redox potential upon interaction with redox partners [67] due to changes in the local pH, polarity of the environment, and interactions with protein amino acid residues and ligands [68]. This can be seen as a particular case of the relevance of changes in the microenvironment of protein prosthetic groups due to local hydrophobicity and pH changes [37,69]. In the bibliography, some C*b*_5_R mutants that retain the FAD group have been reported to present an increase in FAD fluorescence [55]. The tyrosine variants Y93A, D, F, H, and S exhibit alterations in the flavin visible spectra associated with blue shifts in the spectra.

Moreover, in the wild type, P92S, and A, the intrinsic flavin fluorescence was quenched, while some Y93 mutants (substitutions of Y by H, W and A, D, F, and S) exhibited a fluorescent increase, compared with free FAD [55]. Changes in the FAD spectral properties also correlated with shifts up to 20–30 mV in the midpoint potential. The described fluorescence increase in these mutants was lower than that of free FAD or FMN, as we observed for the changes found in the C*b*_5_R autofluorescence and redox potential upon complexing with C*b*_5_ [37]. An explanation for this behavior can be correlated with the existence of two conformational states for FAD: (1) a closed FAD conformation in which the π–π stacking interactions between the adenine and isoalloxazine rings largely quench FAD fluorescence, and (2) a FAD open conformation or extended conformation induced upon binding to nearby peptide side chains. A shift from the close to the open conformation of FAD should produce an increase in flavin fluorescence. A change in the C*b*_5_R’s FAD exposition to the solvent could also help to understand the observed changes in spectroscopic and redox properties [37]. This has also been documented, concerning the structural models obtained by crystallography of the fully reduced and the oxidized form of porcine liver C*b*_5_R that was recently resolved [60]. Local conformational changes in the NADH- and FAD-binding domains were found in C*b*_5_R in the different redox states [60]. This led to the authors suggesting a new role of T66 (T94 in Figure 1) interaction in the release of a proton from the N5 atom of the isoalloxazine ring of FAD, increasing the solvent-accessible surface area of FAD [60]. These results also support that the N5 atom of FAD in C*b*_5_R is stabilized by hydrogen bonding with CαH of Y65 and amide-H of T66 (Y93 and T94 in Figure 1) [66]. The biological function of these changes would prevent reoxidation backflow of the catalytic cycle and the acceleration of the electron transfer to one electron acceptor such as C*b*_5_. It is noteworthy that Y93, T94, and H77 are located at the backside of the FAD group of C*b*_5_R structure when the enzyme is observable in an orientation where the FAD-protruding site is observable (Figure 2 and Figure 3). By computational methods, we found that the FAD-protruding site was associated with the domain where C*b*_5_ docks, and interact with C*b*_5_R (Figure 3a), which contains a significant number of hydrophobic residues; this also helps to rationalize the observed changes on C*b*_5_R’s FAD fluorescence. The distance between the residues implicated in the reduction in electron acceptors is still far from the heme iron group, i.e., the distance from C*b*_5_ heme iron to the NE2 atom of H77 was estimated to be approximately 12 Å (Figure 3a,b). This challenges the role of this residue in the one-electron reduction to the C*b*_5_, although its role and implication in the electron transfer to other redox partners of the C*b*_5_R remain to be experimentally demonstrated.

To obtain answers to some of the opened questions regarding the interaction of C*b*_5_R with ligands, we evaluated the structural features of C*b*_5_R via molecular dynamics by submitting the PDB file of the protein (1UMK) to the dynOmics portal 1.0 server (http://dynomics.pitt.edu/ (accessed on 21 June 2021)) [70].

## 5. Intramolecular Dynamics of C*b*_5_R

Proteins in physiological conditions show a number of motions that help them adapt to intermolecular interactions or accomplish their biological functions [70]. Thus, the intrinsic dynamics are unique and characteristic for each protein. First, we identified the residues with a higher degree of mobility based on the elastic network model 1.0. The image obtained after submission shows a colored image in which the C*b*_5_R structure is colored based on the size of fluctuations driven by the slowest two Gaussian network model (GNM) modes (blue: almost rigid; and red: highly mobile), where the low-frequency modes are highly relative to the biological functions (Figure 3c). We found that the C*b*_5_R areas with a higher degree of mobility define hot motifs centered on the following amino acid residues: Q67 to L80, from V90 to I109, from Lys110 to Q136, T211, A244, H289. In contrast, C*b*_5_R areas with a lower mobility rate are V74, T94, I139, and L205, as indicated by blue-colored motifs (lower mobility), as shown in Figure 3c versus the red-colored motifs that correspond to higher mobility areas.

**Figure 4:**
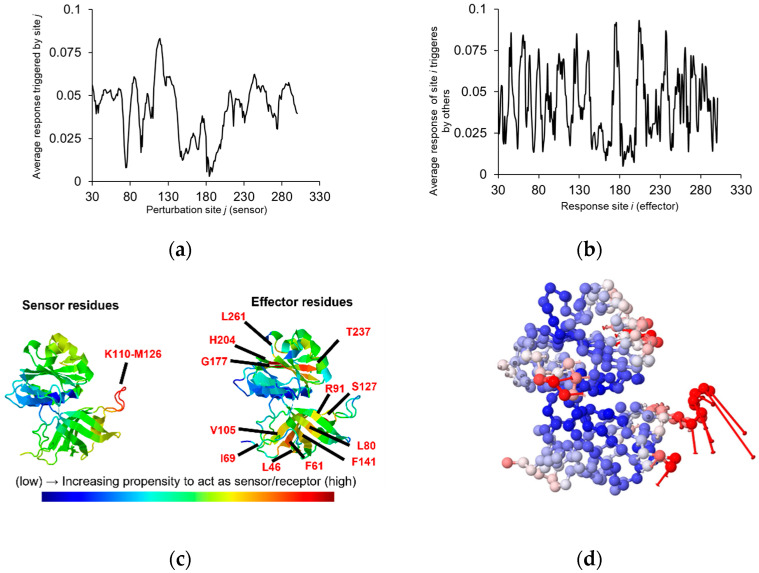
High sensor/effector residues of C*b*_5_R and molecular motions based on compiled information obtained through analysis of the intrinsic dynamics. Correlation between the average response by a site triggered with a perturbation in the C*b*_5_R sequence acting as a sensor is shown in panel (**a**). Correlation between the average response of a site triggered by others with a responsive site in the C*b*_5_R sequence acting as an effector is shown in panel (**b**). Representation of the C*b*_5_R areas, which have an increasing propensity to act as sensor/receptor and label residues located in hot spotted areas, is shown in panel (**c**). Animation obtained with the second-highest ranked model based on the previously analyzed information, where the areas are color coded based on the size of motions where the color indicate the type of fluctuations in a red-white-blue scale (red colors correspond to large fluctuations and blue colors to small fluctuations.), is shown in panel (**d**). Also in this panel, vectors (red lines) represent the direction of motion of each residue.

Regarding the interaction of C*b*_5_R with NADH, crystallographic data obtained with the enzyme in the presence of NADH show differences between C*b*_5_R’s structure in the reduced and the oxidized state and can be correlated with an increase in the solvent-accessible surface area of FAD [69]. These data suggest that C*b*_5_R likely undergoes conformational changes dependent on the interaction with ligands and highlight the existence of protein areas that could sense a structural perturbation such as ligand binding. These areas strongly respond to perturbations through a significant change in their local conformation. In addition, effector areas efficiently communicate perturbations or associated “information” to other sites [70]. We analyzed the presence of sensing and effector areas that could respond to perturbation in certain domains on the C*b*_5_R structure (Figure 4). We determined the existence of a sensing domain in the reductase formed by residues located from K110 to M126 (Figure 4a), which correlated with a loop that contributes to most of the interactions between the FAD-binding domain of the reductase and the adenine dinucleotide moiety of FAD, as previously indicated (Figure 1, yellow background). This area was described as important since the oxygen atoms of the phosphate group of NADH form H bonds with K110 and Y112 and, also, with some of the amino acid residues that are part of the C*b*_5_ interacting domain of C*b*_5_R (F120, G123, K125) [37]. Moreover, several motifs were also detected as effectors, which are susceptible to respond to changes in perturbations associated with sensing areas.

These motifs are centered on L46, F61, I69, L80, R91, V105, K110, S127, F141, G177, H204, T237, L261, L268, A282, and N286, which are amino acid residues labeled in red Figure 4b. The allocation in the structural model of C*b*_5_R of the sensing and effector residues is shown in Figure 4c. Our analysis indicates that some of these residues form part of the effector motifs of C*b*_5_R, which also participate in the interaction with C*b*_5_. These amino acid residues are Y79, R91, and R142, from which R91 was proposed to participate in the tethering of the FAD cofactor by C*b*_5_R [57]. Additionally, through ENM 1.0, a spectrum of motions near their physiological conditions, which often assist in adapting to intermolecular interactions or accomplishing their biological function, can be obtained for C*b*_5_R. In the obtained animated structural model (Figure 4d and Supplementary Figure), those residues implicated in sensing to deliver an effect translated into a fast response are highlighted in red and correlated to those residues which response is slow (blue). By comparing the animation obtained in this figure with that of Figure 3a, which shows the interaction of C*b*_5_R with C*b*_5_ in the same orientation, it can be noted that the binding domain of C*b*_5_ could become altered upon a perturbation acting in the sensing domain to hang the C*b*_5_ molecule. Furthermore, when an NADH molecule interacts with the C*b*_5_R molecule at the NADH-binding lobe K110-M126, a closer interaction between C*b*_5_ and the FAD moiety is promoted. Additionally, these simulations of the C*b*_5_R dynamics allow the comprehension of some mutations effect, such as those found in residues V105M and M126V, which induce methemoglobinemia since they form part of the sensing motif that will cause a substantial alteration in the protein dynamics.

Other suggestions were also proposed for the interaction of C*b*_5_R with ligands modulating the C*b*_5_R function. For example, two reductase conformations were characterized by experiments performed at alkaline pHs, a strategy that we also used to describe the novel enzymatic activities of C*b*_5_ [71,72]. Additionally, a second inactive swollen form in which at least a cysteine and a tyrosine residue are exposed can be stabilized by the addition of mersalyl, a C*b*_5_R inhibitor [28]. These changes support the existence of altered conformations associated with the binding of certain ligands in vivo and by changes in the ionization of the lateral side chain of some amino acid residues.

## Data Availability

The C*b*_5_:C*b*_5_R complex model has been previously published [37]. The data regarding C*b*_5_R Molecular dynamics were obtained by submitting the PDB file of the protein (1UMK) to the dynOmics portal 1.0 server (http://dynomics.pitt.edu/ (accessed on 21 June 2021)).

## Supplementary Materials

The following are available online at https://www.mdpi.com/article/10.3390/ijms23010118/s1.
