# Supplementary figures and images for: Structural Features of Cytochrome b5–Cytochrome b5 Reductase Complex Formation and Implications for the Intramolecular Dynamics of Cytochrome b5 Reductase
Source: Int J Mol Sci. 2021 Dec 23;23(1):118. doi: 10.3390/ijms23010118 (PMC8745658; doi:10.3390/ijms23010118)

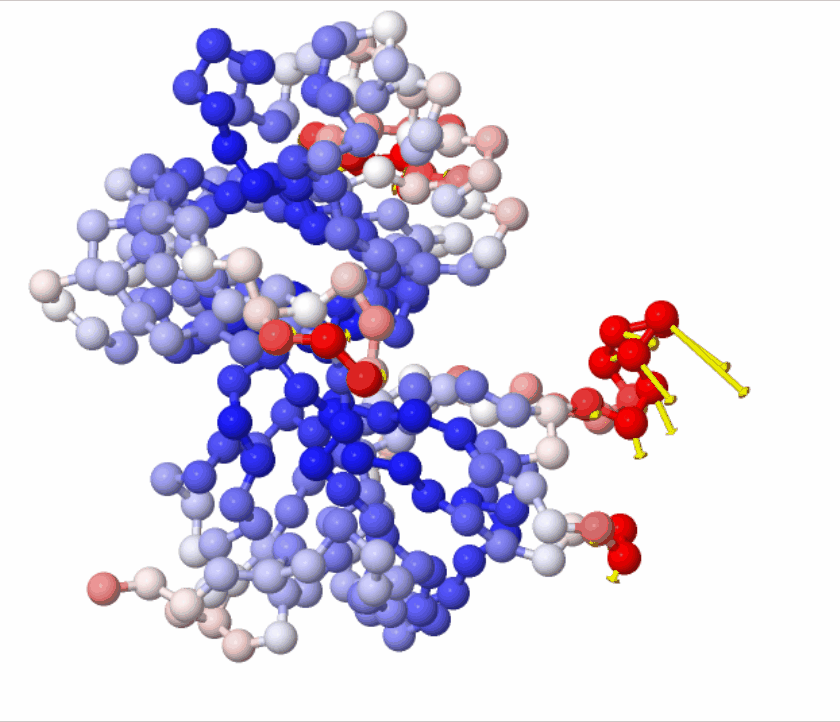

Supplement: Supplementary file 1 [file ijms-23-00118-s001.zip › ijms-1494878-supplementary.gif]
